# A Quick Test of Cognitive Speed: Age‐Related Cognitive Slowing and Screening Thresholds in Turkish‐Speaking Adults

**DOI:** 10.1002/brb3.71767

**Published:** 2026-09-23

**Authors:** Selen Gündüz, Elisabeth H. Wiig, Corvin Melinda

**Affiliations:** ^1^ Department of Child Development, Faculty of Health Science Hacettepe University Ankara Türkiye; ^2^ Professor Emerita of the Department of Communication Sciences and Disorders Boston University Boston Massachusetts USA; ^3^ Knowledge Research Institute, Inc. Arlington Texas USA; ^4^ Department of Speech, Language, and Hearing Sciences, School of Health Professions Texas Tech University Health Sciences Center Lubbock Texas USA

**Keywords:** AQT, cognitive screening, criterion‐referenced assessment, processing speed, Turkish‐speaking adults

## Abstract

**Introduction**: A Quick Test of Cognitive Speed (AQT) is a processing‐speed test that provides objective measures of aspects of executive functioning (attention, working memory, and set shifting) and has been shown to have broad cultural and linguistic applications.

**Objective**: To extend the availability of preliminary sample‐based reference measures of AQT perceptual and cognitive processing speed for use in cognitive screening of Turkish adults. The proposed preliminary sample‐based reference values should be interpreted as preliminary, sample‐based reference ranges rather than clinically validated statistical reference ranges.

**Materials and Methods**: We administered the AQT color, form, and color–form processing‐speed tests to 119 educated, healthy, Turkish‐speaking adults aged 18–80 years. Participants were divided into a younger group (18–55 years; *n* = 53) and an older group (56–80 years; *n* = 66). Data were analyzed using Pearson correlation, linear regression, and one‐way ANOVA following log‐transformation of naming times.

**Results**: In the total group, age correlated significantly (*p* < 0.01) with log‐normal naming times for color (*r* = 0.60), form (*r* = 0.55), and color–form combinations (*r* = 0.63). Linear regression indicated an average increase in naming time of between 1.5 and 2 s per decade. There were significant differences (*p* < 0.01) in naming times for all AQT measures between the younger and older groups, and the effect sizes were large.

**Conclusion**: The present findings provide preliminary sample‐based reference values for perceptual and cognitive processing speed in typically developing Turkish adults and indicate age‐related decline in normal AQT performance. Educational attainment showed a small‐to‐moderate negative association with naming times (*r* = −0.23 to −0.34, *p* < 0.05), suggesting relatively faster performance with higher education. These findings provide preliminary reference values, but require validation in larger, more diverse samples.

## Graphical abstract

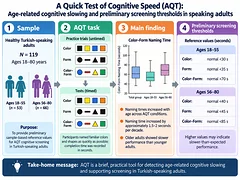

## 1 Introduction

Cognitive aging is often explained by the processing‐speed theory, the central hypothesis of which is that increased age in adulthood is associated with a decrease in the speed with which many processing tasks, especially more complex tasks, can be executed (Salthouse 2004; Silva‐Fernandes et al. 2022). A wide variety of methods exist for measuring cognitive processing speed, but despite these methodological differences, researches indicate that processing‐speed changes vary little across the aging process in neurotypical individuals (Deary et al. 2010; von Krause et al. 2022). The reduction in processing speed leads to impairments in cognitive functioning through two mechanisms: the limited‐time mechanism, which refers to the reduced time available for an individual to process information, and the simultaneity mechanism, which reflects the decreased amount of information that can be maintained simultaneously to complete higher level processing functions (von Krause et al. 2022).

A Quick Test of Cognitive Speed (AQT) is a processing‐speed test that provides objective measures of aspects of executive functioning (attention, working memory, and set shifting) and has broad cultural and linguistic applications (Afshar et al. 2021; Nielsen and Wiig 2006; Petrazzuoli et al. 2014; Takahashi et al. 2012). AQT evaluates processing speed by measuring the time required to complete simple, rapid naming tasks with familiar stimuli (Wiig, Nielsen, Minthon and Warketin 2002). The single‐dimension color and form naming tasks reflect perceptual speed by indexing reaction, retrieval, and response times, whereas the dual‐dimension color–form naming task captures the additional time needed for choice decisions and cognitive set shifting. During this combined task, increased cognitive load redistributes cerebral blood flow toward bilateral posterior temporal–parietal regions while reducing flow to frontal areas (Jalakas et al. 2019). These regions are associated with executive attention, cognitive shifting, visual working memory, and automatic retrieval processes (Turken et al. 2008).

In 2019, the total number of dementia cases identified in individuals aged 65 years and older was 247,727, of whom 150,529 (60.8%) were women. In 2020, the total number of dementia cases identified in this age group was 233,949, with 142,878 (61.1%) of these cases being women (Güner et al. 2024). These numbers may not reflect the actual incidence due to (a) cultural perceptions and stigma that lead many to view dementia not as a medical issue but as a normal part of aging, (b) inadequate or incomplete population statistics for older age groups, and (c) a lack of culturally and linguistically validated assessment tools for dementia—issues documented in recent studies from Türkiye and low‐ and middle‐income countries (Bernstein Sideman et al. 2022; Neugebauer et al. 2025; Polat et al. 2022). These factors motivated us to collect preliminary sample‐based reference data for the AQT color, form, and color–form combination tests from healthy adult Turkish speakers. Our objective was to derive age‐stratified, preliminary sample‐based reference values describing faster and slower performance ranges across a broad age span to support future cognitive screening of both younger and older Turkish adults.

The objectives of this study were pragmatic in nature and sought to extend the availability of norm‐referenced measures of AQT perceptual and cognitive processing speed for use in cognitive screening of Turkish adults. We hypothesized that age would be a significant factor in determining processing speed and that separate norm‐referenced criteria would have to be developed for relatively younger and older (age 55+) Turkish adults. Based on prior research, we expected that sex or years of formal education past Grade 8 would not prove to be significant factors (Nielsen and Wiig 2006; Subirana‐Mirete et al. 2014; Wiig, Nielsen, Minthon and Warketin 2002).

## 2 Materials and Methods

### 2.1 Participants

A total of 124 Turkish adults volunteered to participate in the study. Inclusion criteria required that all participants (a) had completed at least 8 years of formal education; (b) reported no current or previous neurological disorders, neurological trauma, or Type 2 diabetes; and (c) lived independently and were responsible for their own financial affairs. Five adults were excluded based on indications of mild cognitive impairment or relevant medical history. The remaining sample (Group I) consisted of 119 participants—43 females and 76 males—ranging in age from 18 to 80 years. All participants were native Turkish speakers, had completed at least 8 years of formal education, and provided written informed consent in accordance with the Declaration of Helsinki.

Participants were divided into two age‐based subgroups using 55 years as the cutoff. This resulted in a younger group (Group II) of 53 adults aged 18–55 years (*M* = 37.7 years; SD = 12.5; IQR = 27–50 years) and an older group (Group III) of 66 adults aged 56–80 years (*M* = 63.3 years; SD = 6.8; IQR = 58–69 years). The division at age 55 was selected as a pragmatic grouping decision informed by prior AQT studies and the age distribution of the present sample; it was not empirically derived as a clinical or neurobiological threshold. Age was additionally examined as a continuous variable in the correlation and regression analyses.

### 2.2 Materials

**FIGURE 1:**
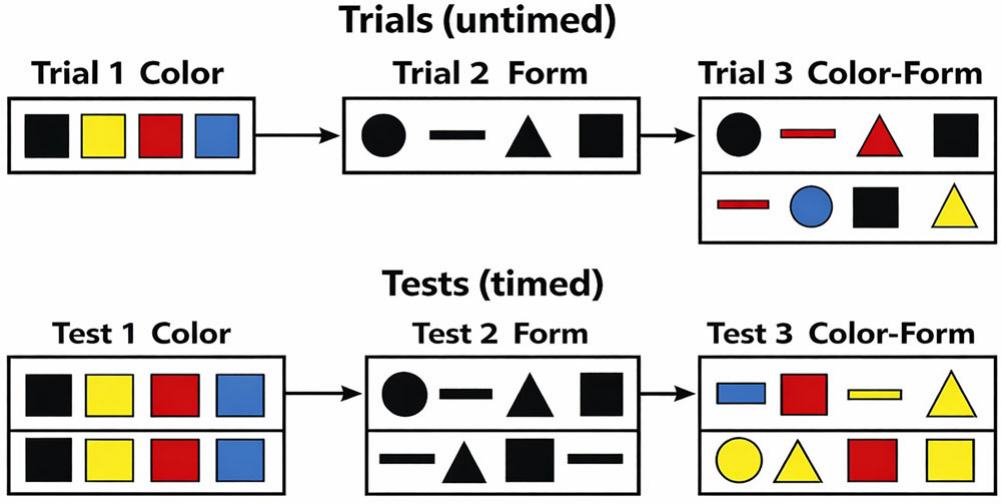
AQT trials and test items for color, form, and color‐form naming.

The AQT color, form, and color–form naming tests (Wiig, Nielsen, Minthon and Warketin 2002) were administered individually in Turkish to obtain preliminary sample‐based reference data. Previous studies have demonstrated that AQT has good test–retest reliability and strong construct validity as a measure of processing speed and executive function (Wiig, Nielsen, Minthon and Warketin 2002; Takahashi et al. 2012; Afshar et al. 2021). Color and form naming require rapid naming of 40 randomly sequenced colored squares (black*/siyah*, blue*/mavi*, red*/kırmızı*, yellow*/sarı*) or 40 black forms (circle*/daire*, square*/kare*, line*/çizgi*, triangle*/üçgen*) (Figure 1). The single‐dimension tests measure reaction, retrieval, and response time (perceptual speed). Color‐form naming (e.g., black circle*/siyah daire*) is a dual‐dimension test that measures cognitive speed (i.e., perceptual speed + overhead from imposed demands on attention, visual working memory, and set‐shifting).

### 2.3 Procedures and Analyses

A speech‐language pathologist, with Turkish as the native language, administered the processing‐speed tests individually. The individual tests were administered in the same order, beginning with a set of trials, followed by color, form, and then color‐form naming. Before the administration of each condition, participants completed a brief practice session to confirm that they could correctly recognize and name all task‐relevant stimuli, including both colors and shapes. Following this familiarization phase, the test was conducted using stimulus cards displaying 40 items arranged in eight rows, each consisting of five stimuli.

In the first condition (AQT1), the stimuli consisted of blue, yellow, red, and black squares; green was intentionally excluded to minimize potential confounding effects related to red–green color vision deficiency. The second condition (AQT2) included black geometric forms—lines, squares, triangles, and circles. The final condition (AQT) required naming combined color–form stimuli derived from the previous tasks (e.g., red square and blue line).

Prior to each task, participants were instructed to verbally name either the color, the shape, or the combined color–shape stimuli, depending on the condition as quickly and accurately as possible. Naming was performed sequentially from left to right and from top to bottom, following a reading‐like order. Completion time for each condition was measured using a digital stopwatch, which was started at the initiation of the first response and stopped once the final stimulus was named. The total administration time for the test was approximately 10 min, and performance scores were defined as the total time in seconds required to complete each condition. Although naming errors were noted during testing, they were not included in subsequent analyses, consistent with standard procedures in rapid automatized naming paradigms (Norton and Wolf 2012).

### 2.4 Statistical Analysis

Descriptive statistics, normality testing, and correlation analyses (Pearson's *r*) were conducted using StatPlus:mac version 5 (AnalystSoft Inc., Walnut, CA, USA) implemented in Microsoft Excel. Normality of continuous variables was assessed using the Shapiro–Wilk (*W*) test. Age and years of education in the total sample (Group I; *N* = 119) were normally distributed. In contrast, the distributions of naming times (in seconds) deviated significantly from normality in the total group (Group I; age 18–80), Group II (age 18–55), and Group III (age 56–80). Therefore, all naming time variables were logarithmically transformed (natural logarithm [ln]) prior to inferential statistical analyses. To examine the association between age and color–form naming performance in the total group, a linear regression analysis was performed with age (years) as the independent variable and ln‐transformed naming times as the dependent variable. Group differences were examined using one‐way ANOVA. Preliminary, sample‐based reference ranges were derived using standard deviation–based descriptive criteria (+1 SD and +2 SD). Statistical significance was set at *p* < 0.05.

## 3 Results

We examined the associations between age and *ln*‐transformed color, form, and color–form naming times in the total sample. Pearson correlations showed age was moderately associated with ln color (*r* = .61) and ln color–form (*r* = 0.66) and weakly associated with ln form (*r* = 0.59) naming times (all *p* < 0.01; Group I, *n* = 119). Age accounting for 37% of the variance in color naming, 35% in form naming, and 44% in color–form naming performance.

To further investigate the relationship between age and color–form naming in the total group, a linear regression analysis was conducted with age (years) as the independent variable and ln‐transformed color–form naming time as the dependent variable. The regression model was statistically significant, *F* = 91.67, *p* < 0.01. The resulting regression equation is given as:

$$\ln\left(\mathrm{CF}\right)=3.5802+0.0106\times \mathrm{age}$$

The regression coefficient (0.0106) corresponds to an approximate multiplicative increase in raw naming time, which translates to an estimated increase of ∼1.5–2 s per decade based on back‐transformation. This model indicates an intercept corresponding to approximately 36 s and an estimated increase in color–form naming time of approximately 1.5–2 s per decade.

The correlation between years of education and ln color (*r* = −0.23) and form (*r* = −0.34) proved significant (*p* < 0.05), and the associated effect sizes were weak. Thus, years of education contributed to 5% of the variance for color and 12% for form naming. In contrast, the correlation between years of education and *ln* color‐form naming (*r* = −0.07) proved non‐significant (*p* > 0.05).

We additionally examined the effect of sex on AQT naming performance. One‐way ANOVA revealed no significant differences between males and females in mean naming times for color (*F* (1, 117) = 1.94, *p* > 0.05), form (*F* (1, 117) = 0.97, *p* > 0.05), or color–form naming (*F* (1, 117) = 3.26, *p* > 0.05).

**FIGURE 2:**
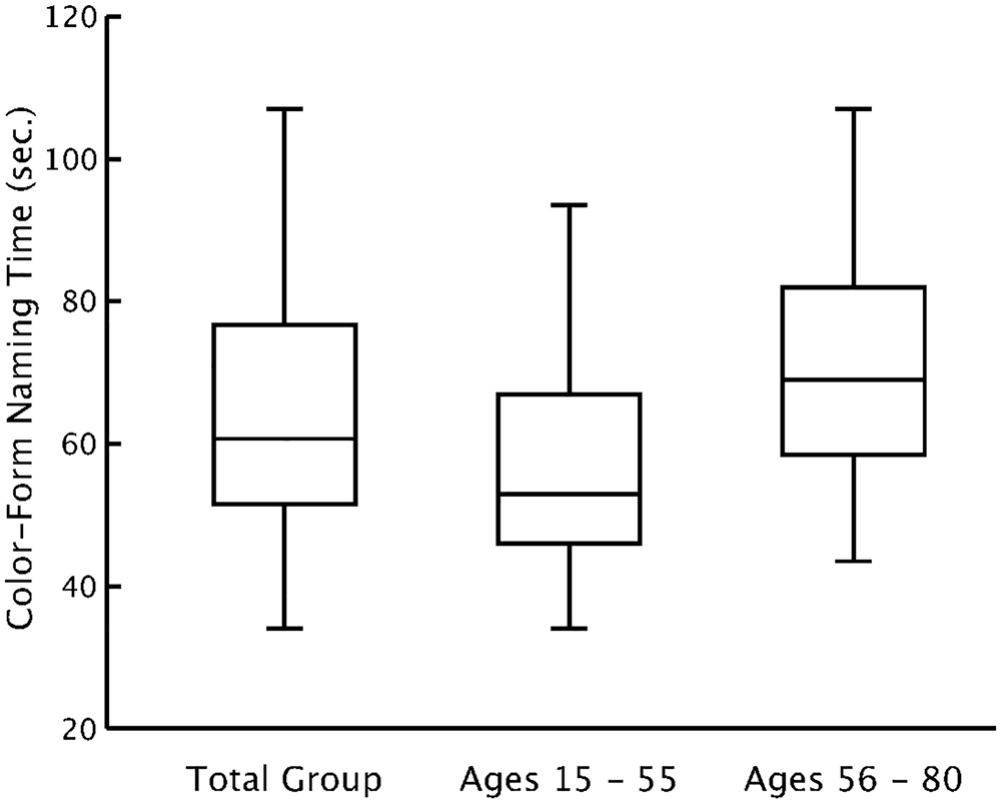
Box plots for color‐form naming times for Groups I, II, and III.

Descriptive statistics for color, form, and color‐form naming times (in seconds) for Groups I, II, and III are reported in Table 1. One‐way ANOVA indicated significant mean differences between Group II and III for color (*F*_1, 117_ = 26.58; *p* < 0.01; *η* ^2^ = 0.19), form (*F*_1, 117_ = 20.72; *p* < 0.01; *η* ^2^ = 0.15) and color‐form naming (*F*_1, 117_ = 28.3; *p* < 0.01; *η* ^2^ = 0.19). All effect sizes were large. Figure 2 shows box plots for color‐form naming times for Groups I, II, and III. The plots clearly show that the distributions for color‐form naming were left skewed, as were the distributions for color and form naming. Naming errors were recorded, but because participants in Groups II and III made between zero (0) and a maximum of three (3) errors on any measure, naming errors were not further analyzed for criterion referencing.

**TABLE 1:** AQT processing‐speed variables (in seconds) for the total group (Group I) (*N* = 119), Group II (age 18–55), and Group III (age 56–80).

AQT	Group I (*n* = 119)		Group II (*n* = 53)		Group III (*n* = 66)	
	*M*	SD	*M*	SD	*M*	SD
Color (s)	27.38	7.39	23.96	6.49	30.13	6.94
Form (s)	30.35	8.77	26.75	6.98	33.25	9.03
Color‐form (s)	64.34	16.68	56.57	14.67	70.58	15.62

*Note*: M: mean; SD: standard deviation.

We calculated intercorrelations (*r*) among the ln naming time measures in the total group (Group I) to evaluate the degree to which single‐dimension naming (color and form) contributed to dual‐dimension (color‐form) naming time. The correlations between ln color and form (*r* = 0.80), color and color‐form (*r* = 0.81), and form and color‐form naming (*r* = 0.79) all proved significant (*p* < 0.01), and effect sizes were large. The results indicate that color and form naming times contributed equally and with large effects to color‐form combination times.

We used standard deviations to derive preliminary, sample‐based reference ranges describing expected and slower naming times. The sample‐based reference values for color, form, and color‐form naming in Group II (18–55 year) and III (56–80 year) were constructed by using the respective standard deviations. For descriptive purposes, +1 SD above the mean was used as the upper boundary of the expected performance range. Values between +1 and +2 SD were classified as slower‐than‐expected, and values at or above +2 SD as markedly slower than the sample mean; these categories are descriptive and should not be interpreted as clinically validated thresholds. The respective values (in seconds) were rounded to the nearest whole number for ease of administration. The proposed preliminary sample‐based reference values for Group II and III are shown in Table 2.

**TABLE 2:** Criterion‐referenced cut‐off times for the normal, slower‐than‐normal, and atypical/pathological performances ranges for age 18–55 and 56–80 years.

Age groups	Normal range	Slower‐than‐normal	Atypical/pathological
Age 18–55			
Color	< 30 s	31–34 s	> 35 s
Form	< 35 s	36–39 s	> 40 s
Color‐form	< 70 s	71–84 s	> 85 s
Age 56–80			
Color	< 40 s	41–44 s	> 45 s
Form	< 45 s	46–49 s	> 50 s
Color‐form	< 85 s	86–99 s	> 100 s

## 4 Discussion

The study provided data for deriving preliminary, sample‐based reference values for the AQT color, form, and color‐form tests based on the performance of a culturally and linguistically representative sample of Turkish speakers of age 18– 80 years. Previously, criterion cut‐off time scores have been developed for these AQT processing‐speed tests for English (Wiig et al. 2002), Swedish (Classon et al. 2022; Wiig et al. 2003), Italian (Petrazzuoli et al. 2014), Spanish (Subirana‐Mirete et al. 2014), Arabic (Wiig and Al‐Halees 2013), Japanese (Takahashi et al. 2012), and Persian (Afshar et al. 2021).

In this study, age had a large effect on the naming times for colors, forms, and color‐form combinations. The size of the correlations (*r*) between age and ln naming times ranged from *r* = 0.66 to 0.59 among the Turkish speakers, indicating that age accounted for between 35% and 44% of the variance. The contribution of age to the AQT naming times observed for the Turkish speakers is similar to that of Spanish speakers (35.6%–39.4%) (Subirana‐Mirete et al. 2014). It is larger than reported for older Arabic (27%–29%) and Italian speakers (19%–26%) (Petrazzuoli et al. 2014; Wiig and Al‐Halees 2013). The contribution of age to AQT naming times by Turkish speakers is also considerably larger than that reported for English speakers in the same age range (8%–19%) (Wiig et al. 2007). Cohort‐related differences in educational background and lifelong reading automatization may further amplify age effects in these populations. Cultural and cognitive reserve–related factors, including variability in educational attainment and daily engagement in speeded visual–verbal tasks, may also contribute to the observed differences. Finally, methodological variations across studies, including age range definitions and sample characteristics, should be considered when interpreting cross‐linguistic differences in the magnitude of age‐related effects on AQT performance. The increase in color‐form naming time as a function of age of 1.5– 2 s per decade for Turkish speakers compares to an increase of 1 s per decade reported for English speakers in the age range from 15 to 85 years (Jacobson et al. 2004; Wiig et al. 2007). However, it is less than the 4 s per decade increase reported for Hispanic speakers in the same age range (18–85 years) (Subirana‐Mirete et al. 2014). The relatively slow increase in naming times over the age range between 18 and 80 years and the age ranges subject to effects of dementia caused us to select age 55 as the dividing point between a younger and an older group. Sex proved to be a non‐contributing factor for naming times, and this concurs with previous findings (Afshar et al. 2021; Petrazzuoli et al. 2014).

Previous studies have shown that years of education exert a minimal influence on AQT naming times once basic literacy has been achieved (Grade 8 or higher) (Nielsen and Wiig 2006; Subirana‐Mirete et al. 2014; Wiig and Al‐Halees 2013; Wiig et al. 2002; Wiig et al. 2003). In the present study, all Turkish adult participants had completed at least 8 years of formal education; therefore, we initially hypothesized that years of education would not have a significant effect on AQT naming performance. Contrary to this expectation, years of education demonstrated a small negative effect on color naming times and a moderate effect on form naming times, indicating faster performance with increasing educational attainment. In contrast, the hypothesis was supported for color–form combination naming, as years of education did not significantly influence naming times. These findings may reflect differences in educational practices or instructional emphasis rather than true intra‐individual or cross‐linguistic differences between Turkish and English speakers.  Educational attainment may be associated with relatively better preservation of perceptual and cognitive processing speed in adulthood.

Comparisons of ln naming‐time means indicated significant differences between the younger (Group II) and older (Group III) speakers for all AQT measures. The effect sizes were large (*η* ^2^ = 0.15–0.19), indicating substantial age‐group differences in naming performance. Inter‐individual variations in processing speed were reflected by standard deviations that were approximately one fourth the size of the mean. Intra‐individual factors may relate to general health or other conditions.

Because color‐form naming is the measure most commonly used for screening and monitoring cognitive status, we were interested in the relative contributions of single–dimension processing speed measures (color, form) to the dual‐dimension measure (color‐form). Intercorrelations between the measures color and color‐form (*r* = 0.81) and form and color‐form (*r* = 0.79) indicated that color and form naming contributed equally to and had large effects on color‐form combination naming.

The fact that the number of naming errors proved to be very low with three errors as the maximum for color‐form naming corresponds to previous findings for, among others, Arabic and English speakers (Wiig and Al‐Halees 2013; Wiig et al. 2002). On the AQT color‐form naming measure, the maximum number of errors for Arabic and English speakers, for example, was three (*M* = 0.49). In clinical practice, the number of self‐corrected or uncorrected naming errors may be recorded for the AQT color‐form naming measures to provide an index of self‐monitoring.

The current sample consisted of relatively well‐educated, cognitively healthy, and independently living individuals, which was an intentional choice, as reference values for cognitive screening tools such as the AQT are typically derived from healthy populations. Consistent with prior research, sex and education beyond a minimum threshold are not expected to significantly influence AQT performance (Nielsen and Wiig 2006; Wiig et al. 2002; Subirana‐Mirete et al. 2014). However, this relatively homogeneous sample may limit generalizability to broader populations, and the current findings should therefore be considered preliminary reference data. Another limitation is that the upper limit of the age range was at age 80 years. The expected life span of adults has increased globally, and future studies of Turkish adults may need to include validated AQT reference values for adults of more advanced ages. While cross‐cultural comparisons are informative, the present study does not allow for direct testing of underlying cultural or cognitive mechanisms. Therefore, these interpretations should be considered tentative and require further empirical validation. Future studies employing cross‐cultural designs and comparable methodologies are needed to more directly examine the potential mechanisms underlying these observed differences.

## 5 Conclusion

Considering the critical importance of early diagnosis and intervention, the availability of preliminary Turkish reference data for the AQT may support future research and, following clinical validation, may contribute to cognitive screening in Turkish‐speaking adults. Adhering to international guidelines in clinical settings enhances the cultural equivalence of assessment procedures, and the development of equivalent assessment tools across languages facilitates cross‐cultural and cross‐national information exchange. In this context, AQT preliminary reference data may provide a useful foundation for future validation studies for Turkish‐speaking adult and elderly populations. The AQT color, form, and color–form naming measures are brief, easy to administer (3–5 min), and straightforward to interpret by trained personnel, supporting timely referral to medical professionals. Moreover, these measures are highly reliable and have demonstrated sensitivity to cognitive impairment and early treatment response in Alzheimer's disease (Takahashi et al. 2012). Overall, the present findings provide preliminary reference values for perceptual and cognitive processing speed in typically developing Turkish adults and indicate moderate to large age‐related decline in normal AQT performance.

## Author Contributions

**Selen Gündüz**: Methodology, data curation, investigation, visualization, formal analysis, resources, writing – original draft. **Elisabeth H. Wiig**: Conceptualization, methodology, formal analysis, writing – review and editing, and supervision. **Corvin Melinda**: Conceptualization, methodology, writing – review and editing, and supervision.

## Funding

The authors have nothing to report.

## Ethics Statement

This study was approved by the Texas Tech University Health Sciences Center Institutional Review Board for The Protection of Human Subjects (Approval No: L12‐143). Informed consent was obtained from all individual participants included in the study.

## Conflicts of Interest

The authors declare no conflicts of interest.

## Data Availability

The data that support the findings of this study are available from the corresponding author upon reasonable request. Due to ethical and privacy considerations, the data are not publicly available as they contain information that could compromise participant confidentiality.
